# Synthesis and Properties of Highly Tilted Antiferroelectric Liquid Crystalline (*R*) Enantiomers

**DOI:** 10.3390/ma17204967

**Published:** 2024-10-11

**Authors:** Magdalena Urbańska, Monika Zając, Michał Czerwiński, Przemysław Morawiak, Alexej Bubnov, Aleksandra Deptuch

**Affiliations:** 1 Institute of Chemistry, Military University of Technology, ul. Sylwestra Kaliskiego 2, 00-908 Warsaw, Poland; monika.zajac@wat.edu.pl (M.Z.); michal.czerwinski@wat.edu.pl (M.C.); 2 Institute of Applied Physics, Military University of Technology, ul. Sylwestra Kaliskiego 2, 00-908 Warsaw, Poland; przemyslaw.morawiak@wat.edu.pl; 3 Institute of Physics of the Czech Academy of Sciences, Na Slovance 1999/2, 18200 Prague, Czech Republic; bubnov@fzu.cz; 4 Institute of Nuclear Physics Polish Academy of Sciences, Radzikowskiego 152, 31-342 Krakow, Poland; aleksandra.deptuch@ifj.edu.pl

**Keywords:** (*R*) enantiomer, synthesis, HPLC, SmC_A_* phase, helical pitch, tilt angle, eutectic mixtures, X-ray diffraction

## Abstract

This work reports the synthesis method and various properties of four rod-like antiferroelectric (*R*) laterally substituted enantiomers, with or without fluorine atoms used as substituents in the benzene ring. The influence of fluorine substitution on the mesophase temperature range was determined. The synthesized compounds are three-ring rod-like smectics with a chiral center based on (*R*)-(−)-2-octanol. Their chemical and optical purity was checked using high-performance liquid chromatography (HPLC). Two newly synthesized enantiomers and three previously reported (*R*) enantiomers were used to formulate two antiferroelectric mixtures. The mesomorphic behavior was characterized by polarizing optical microscopy, differential scanning calorimetry, and X-ray diffraction (XRD). The helical pitch and tilt angle measurements were done using the selective light reflection phenomenon and the electro-optical method, respectively. All the enantiomers exhibit a wide temperature range of the antiferroelectric phase, with a high tilt angle. Furthermore, the enantiomer with lateral fluorine substitution in the ortho position has a very long helical pitch (more than 2.0 µm), relatively low enthalpy of melting point, and a tilt angle close to 45 degrees. The designed (*R*) enantiomers can be useful for formulating eutectic mixtures for further use in various devices, including photonics and optoelectronics.

## 1. Introduction

In smectic liquid crystalline (LC) phases, the molecules exhibit translational order in a layered structure, with at least short-range positional order within layers [1,2]. The formation of chiral smectic LC phases results from the ordered propagation of the tilt angle, layer by layer, so the assembly of molecular units adopts the helical arrangement on a macroscopic level [3,4]. Since the first reports on chiral synclinic (ferroelectric) [5] and chiral anticlinic (antiferroelectric) [6] LCs appeared, significant research efforts have continued to design chiral LC systems [7,8,9,10,11]. Due to the presence of a stereocenter in the molecular unit, mesogens can mainly adopt a helical conformation. In this way, molecular-level chirality is transferred to the macroscopic scale in the LC phase. This leads to exciting applications in advanced displays, sensors, nonlinear optical devices, organic photovoltaic cells, and others [4,12,13,14,15,16,17,18,19,20,21,22,23,24]. The most promising LC materials for practical use in display technology possess the SmC_A_* phase with a tilt angle close to 45° [25,26]. Such antiferroelectric liquid crystals (AFLCs) have orthoconic properties [27,28,29,30] and are described as OAFLCs—orthoconic antiferroelectric liquid crystals. In the surface stabilized geometry (SSOAFLCs), these materials can be considered as an optically negative medium and, at the same time, optically isotropic for a beam of light at normal incidence. This is due to the optical axis of the refractive medium being aligned along the plane of the smectic layer and perpendicular to the boundary surfaces, making it collinear with the light passing through the display at normal incidence. Such SSOAFLC retains all the advantages of AFLC and, most importantly, achieves extremely high optical contrast due to the almost perfectly dark state at zero electric field. This occurs because optical defects remain invisible between crossed polarizers, regardless of any local disorientation of smectic layers that is normal within the SSOAFLC structure. Recent extensive studies have shown a significant influence of the molecular structure of AFLCs on the formation of their orthoconic behavior [31,32,33,34,35,36,37,38,39,40,41]. These properties are typically exhibited by three-ring compounds, often containing fluorine atom(s) as lateral substituents in the benzene ring, with a terminal perfluoroalkoxylalkoxy or perfluoroalkanoyloxyalkoxy chain complemented by various combinations of chirality centers. While (S) enantiomers are most commonly used, several (*R*) enantiomers have recently been synthesized [42,43]. Mixing (*R*) and (*S*) enantiomers results in OAFLC mixtures with a very long helical pitch [44], which is one of the critical parameters for OAFLC applications [45]. The helical pitch for most OAFLCs is shorter than 1.0 μm, and a very thin cell is necessary to obtain a well-oriented smectic layer. Thus, LC materials with a long helical pitch are highly sought.

Here, we present the synthesis of four new (*R*) enantiomers, complemented by a detailed investigation of their chemical and physical properties and potential application in the formulation of mixtures, as they are of high practical importance. This work demonstrates that some synthesized LC materials exhibit near-orthoconic behavior simultaneously with an exceptionally long helical pitch. Further research will undoubtedly focus on their utilization as functional materials in novel multicomponent LC mixtures. So far, only a few mixtures containing (*R*) enantiomers have been formulated. Hence, these materials are undoubtedly a novelty in the group of chiral antiferroelectrics.

## 2. Materials and Methods

### 2.1. Method for the Synthesis of (R) Enantiomers

**Figure 1:**
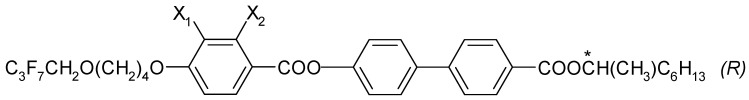
General chemical formula of (*R*) enantiomers; (*) indicates the chiral center.

The chemical formula (in general form) for the four esters that were designed and synthesized with (*R*) configuration is presented in Figure 1. These (*R*) enantiomers have the acronym **I.4.(X_1_X_2_) (*R*)**, where **X_1_**, **X_2_** = **H** or **F**.

**Figure 2:**
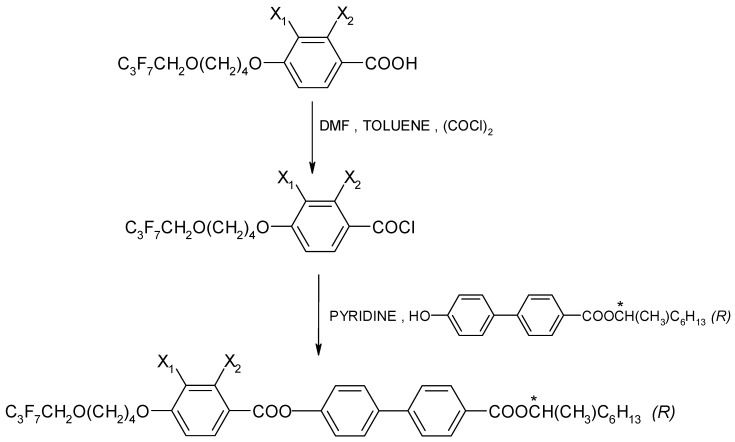
General scheme for the synthesis of (*R*) enantiomers; (*) indicates the chiral center.

The enantiomers were synthesized by treating a chiral phenol with benzoic acid chloride in the presence of pyridine, as shown in Figure 2. An efficient preparative method was used to synthesize a chiral hydroxyester of high optical purity [46]. The synthesis was carried out as described in Ref. [47]. The course of the reaction was explained using the example of the enantiomer **I.4.(HH) (*R*)**, which is presented in the Supplementary Materials.

The chemical purity of the synthesized enantiomers was checked first by thin-layer chromatography (there was always one spot on the plate) and then by high-performance liquid chromatography. HPLC-PDA-MS (APCI-ESI dual source) Shimadzu LCMS 2010 EV equipped with a polychromatic UV-VIS detector (Shimadzu Co., Kyoto, Japan) was used to check the purity, and the purity was over 99% for all (*R*) enantiomers. The results of the ionization process of the analyzed enantiomers are presented in Table 1. The MS data and the corresponding purity curves for these enantiomers are shown in Figures S1–S4 (see Supplementary Materials).

### 2.2. Determination of Optical Purity of (R) Enantiomers

Amylose-based polysaccharide derivatives [48] are the most effective CSPs for enantioseparation of this type of smectic [49,50]. However, in this case, an equally effective immobilized brush-type column with π-electron donor and π-electron acceptor groups was chosen to test the optical purity. Acetonitrile/water 99/1 (*v*/*v*) was used as a mobile phase. Isocratic elution was used in the analysis. The enantiomers were dissolved in ACN at 0.6 mg·mL^−1^; the injected sample was 5 µL. HPLC-grade ACN (min. 99.9%) was purchased from a Polish company (POCH S.A., Gliwice, Poland). Double-distilled water was taken (a glass distiller, GFL 2302 model, manufactured in Germany, was used for distillation).

**Figure 3:**
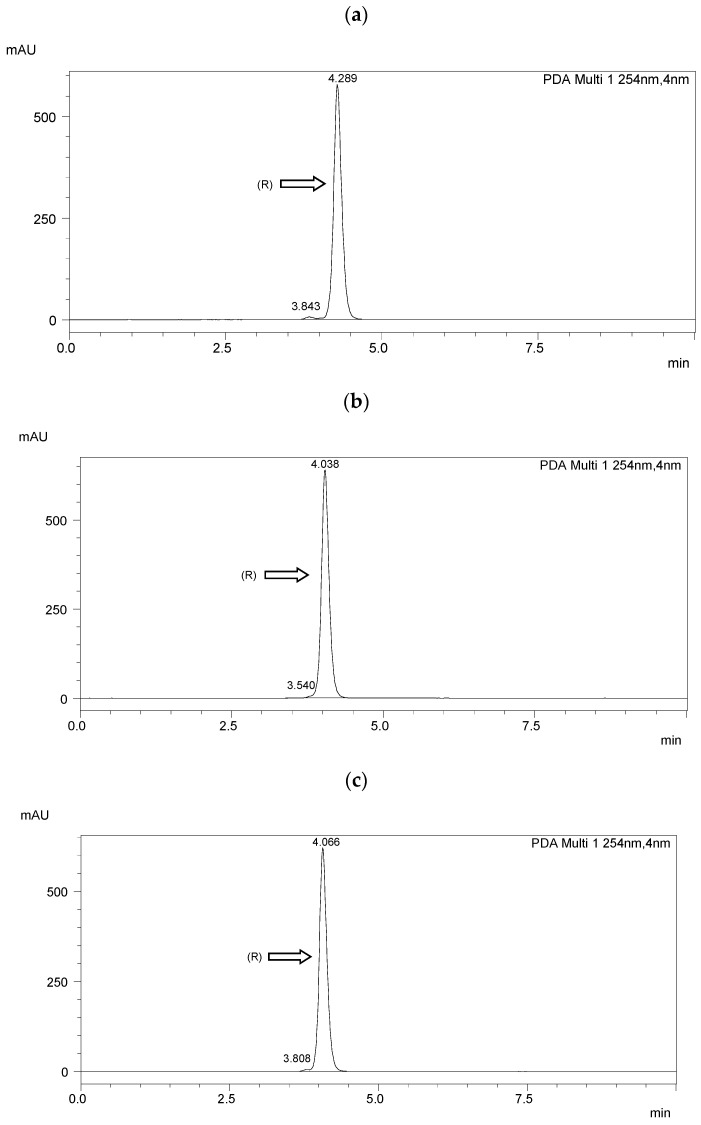

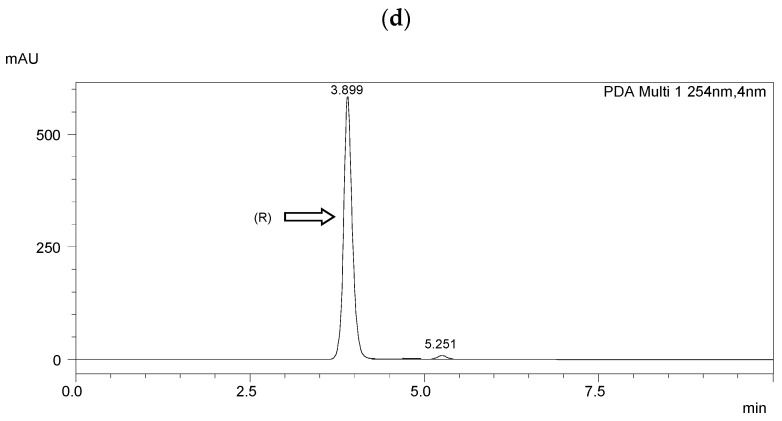
HPLC chromatograms of the enantioseparation of (*R*) enantiomers shown in figures: (**a**) **I.4.(HH) (*R*)**; (**b**) **I.4.(HF) (*R*)**; (**c**) **I.4.(FH) (*R*)**; (**d**) **I.4.(FF) (*R*)**.

Chiral separation was performed using a Shimadzu LC-20AP HPLC system (Shimadzu Co., Kyoto, Japan). A diode array detector (SPD-M20A) was used. Data were collected using Shimadzu software (Labsolutions, 2010-2017 Shimadzu Co.). The measurements were performed at room temperature (25 °C). The flow rate of the mobile phase was 1.0 mL·min^−1^. The detection wavelength was 254 nm. A ReproSil Chiral NR column with a particle size of 5 μm, surface area of 350 m^2^·g^−1^, dimensions of 250 mm × 4.6 mm i.d, and a pore size of 100 Å (Dr. Maisch, Ammerbuch, Germany) was taken for the enantioseparation. The results of HPLC separation are presented in Figure 3 and Table 2. The resolution (R_s_) [51] was higher than 1.5 in each case, indicating a baseline separation.

The optical purity of the synthesized (*R*) enantiomers is high, especially for the monofluorinated enantiomers *ee* > 99%. If recrystallized again from anhydrous ethanol, their purity would increase even more, especially with the difluorinated enantiomer. The optical purity of the chiral biphenol used was more than 98%.

### 2.3. Experimental Methods

The mesophases sequence was determined by polarizing optical microscopy (POM) using an Olympus BX51 polarizing microscope (Shinjuku, Tokyo, Japan) equipped with a Linkam heating/cooling stage THMS-600 (Linkam Scientific Instruments Ltd., Tadworth, UK).

The phase transition temperatures were also precisely determined by differential scanning calorimetry (DSC) using a Perkin-Elmer DSC8000 calorimeter (PerkinElmer, Shelton, CT, USA). The samples of ~3–7 mg, hermetically sealed in aluminum pans, were placed in a nitrogen-filled calorimeter chamber. Temperature and enthalpy change values were calibrated on the extrapolated onset temperatures and the enthalpy changes of the melting points of water, indium, and zinc. The calorimetric measurements were performed on cooling/heating cycles at a rate of 5 K·min^−1^.

The helical pitch [p] was determined on homeotropically aligned samples of materials placed on a single glass plate while leaving the other surface of the sample free. The measurements of the helical pitch were based on the selective light reflection phenomenon [52]. The p(T) characteristics were calculated from the equations p = λ/n for the SmC_A_* phase and p = λ/2n for the SmC* phase (the value of the average refractive index n = 1.5 was estimated according to Ref. [53]). The transmission spectra were acquired using a Shimadzu UV-VIS-NIR spectrometer (wavelength range of 360 nm–3000 nm). AMLWU7 controller with Peltier element was used for temperature control within the 2–110 °C range and accuracy ± 0.1 K. All measurements were performed in the cooling cycle.

The polarimetry method determined the helical twist sense for two chiral phases [54]. The helical twist sense inversion temperature was determined by analyzing the temperature-dependent transmission of the light through a homeotropically aligned sample under a polarizing optical microscope; the brightest texture indicates an unwound state [34,55].

The tilt angle of the director (Θ) was determined optically using well-aligned planar samples at the bookshelf-like surface stabilized structure by observing the difference between extinction positions at crossed polarizers under opposite d.c. electric fields ±40 kV·cm^−1^. All the measurements were done during the cooling cycle.

The X-ray diffraction method − X’Pert PRO diffractometer (Malvern PANalytical, Malvern, Worcestershire, UK) with a TTK-450 temperature attachment (Anton Paar, Graz, Austria) was used to determine the smectic layer spacing [56]. Before the measurement, the samples were heated until an isotropic liquid was obtained, and then the diffraction patterns were collected in the 2θ = 1.7–30° range (CuKα radiation, Bragg-Brentano geometry) during cooling. Data analysis was performed in WinPLOTR (version from April 2019) [57] and OriginPro 2020b.

## 3. Results

### 3.1. Mesomorphic Behavior

In the case of the synthesized liquid crystalline (*R*) enantiomers, the phase sequences were determined based on the characteristic textures and their changes observed in POM. The phase transition temperatures and transition enthalpies were estimated with high accuracy from DSC measurements. The phase sequences and phase transition temperatures, measured during cooling, melting points, m.p., and clearing points, c.p., measured during heating and respective phase transition enthalpies, ΔH, obtained by DSC for (*R*) enantiomers are summarized in Table 3.

**Figure 4:**
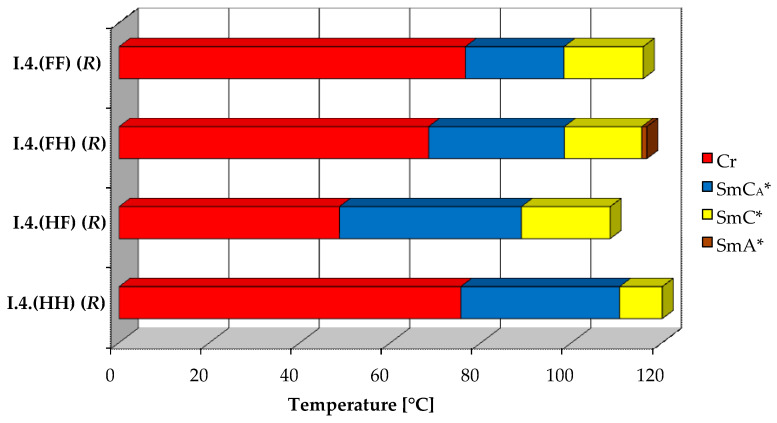
Visualization of phase temperature ranges of (*R*) enantiomers.

**Figure 5:**
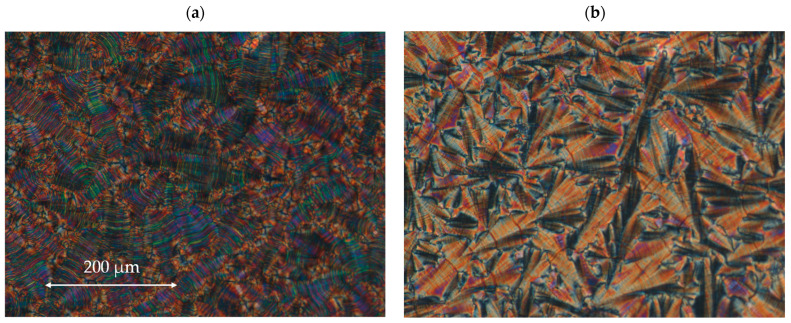
The microphotographs of the microscopic pattern for the enantiomer **I.4.(HH) (*R*)** observed during the cooling cycle: (**a**) in the SmC* phase at 121.1 °C and (**b**) in the SmC_A_* phase at 96.7 °C. The width of the microphotographs is ~600 μm.

All four designed enantiomers exhibit the ferroelectric (SmC*) and the antiferroelectric (SmC_A_*) phases. The paraelectric orthogonal (SmA*) phase is observed in a very short temperature range only for the enantiomer **I.4.(FH) (*R*)**. The polar, synclinic SmC*, and anticlinic SmC_A_* phases occur over a reasonably broad temperature range. The broadest temperature range of the antiferroelectric phase (above 40 °C) is observed for the monofluorinated enantiomer with **(HF)** type of substitution. The highest clearing point was detected for the unsubstituted enantiomer. The lowest melting and clearing points were detected for enantiomer **I.4.(HF) (*R*)**, see Figure 4. Characteristic microscopic patterns for the SmC_A_* and SmC* phases are presented in Figure 5a,b. Both the ferroelectric and antiferroelectric phases possess broken fan-shaped textures, and the differences between them are shown for the enantiomer **I.4.(HH) (*R*)**.

All the SmC_A_*−SmC* transitions exhibit low enthalpy values equal to or below 0.07 J·g^−1^. The SmC* to SmA* phase transition has a higher enthalpy (0.8 J·g^−1^). The highest enthalpy for the clearing point is estimated for the unsubstituted enantiomer—**I.4.(HH) (*R*)**. Melting point enthalpies are pretty low for the compounds **I.4(HF) (*R*)** and **I.4(HH) (*R*)**, while they are significantly higher (greater than 30 J·g^−1^) for the remaining compounds.

The sequence of mesophases was the same when comparing the (*S*) [31] and (*R*) enantiomers. Slightly lower clearing points were observed for (*S*) enantiomers. The source of the differences in phase transition temperatures for (*S*) and (*R*) enantiomers may be these compounds’ slightly different enantiomeric purity.

### 3.2. Helical Pitch and Twist Sense

**Figure 6:**
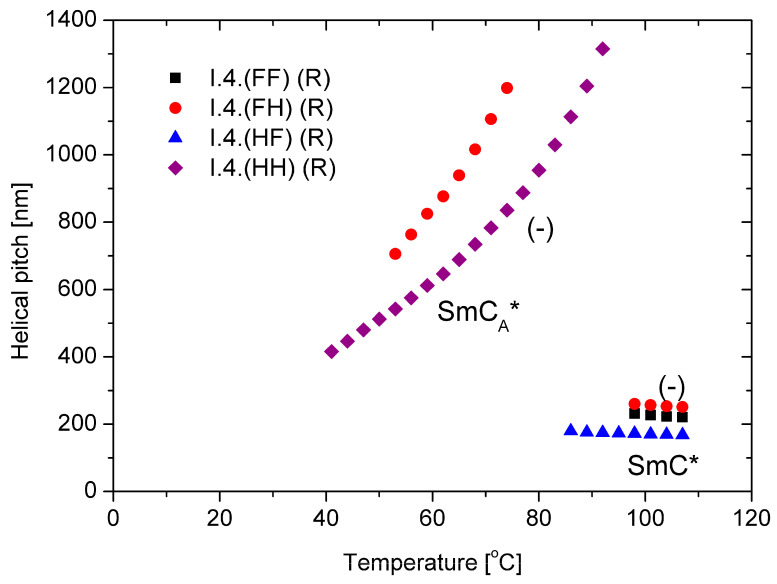
Temperature dependence of the helical pitch length for (*R*) enantiomers; “-” indicates the left-handed helix.

The behavior of the main parameter of the helical structure, namely, the helical pitch versus temperature, is presented in Figure 6 for (R) enantiomers. Within the ferroelectric SmC* phase, the helical pitch remains almost temperature-independent and does not exceed 300 nm with a left-handed helix. The helix is left-handed in the antiferroelectric phase, but the pitch length increases with temperature. The enantiomer with a fluorine atom in the meta position exhibits a longer helical pitch in all measuring ranges than the unsubstituted enantiomer. For the enantiomers **I.4.(HF) (R)** and **I.4.(FF) (R)**, the helical pitch in the anticlinic SmC_A_* phase is out of the measuring range (i.e., above 2.0 µm) of the used spectrophotometer.

### 3.3. X-ray Diffraction

**Figure 7:**
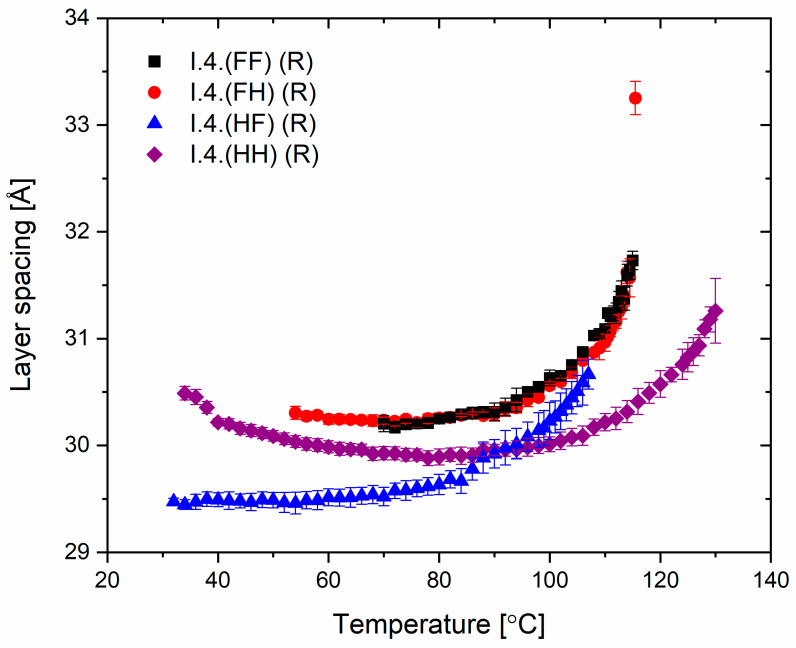
The smectic layer spacing of (R) enantiomers versus temperature.

The spacing between the smectic layers as a function of temperature for all enantiomers is shown in Figure 7. The layer spacing for the orthogonal SmA* phase was observed only for a single temperature, while in the SmC* and SmC_A_* phases, the layer spacing values decreased with decreasing temperature. The layer shrinkage at the SmA*−SmC* transition for the enantiomer **I.4.(FH) (R)** equals ca. 5%. For the enantiomer **I.4.(HH) (R)**, the layer spacing slightly increases just before crystallization, which may be related to the transition to another phase (named hexatic), previously suspected for the enantiomer **I.4.(HH) (S)**.

Most importantly, from an application perspective, layer thickness studies confirm the mesomorphic studies, which show a direct phase transition from the isotropic phase to tilted smectic phases in three of the four enantiomers. This crucial characteristic helps minimize defects in surface-stabilized AFLC geometries [35], improving the optical quality of devices based on such LC materials.

### 3.4. Tilt Angle

**Figure 8:**
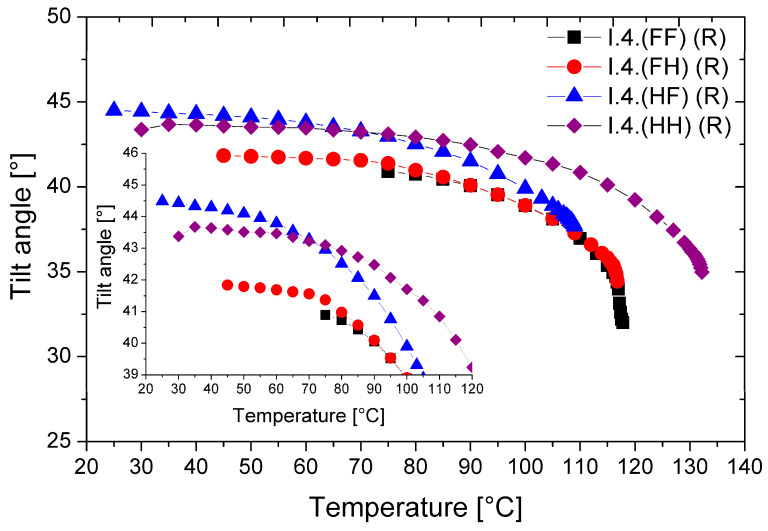
Temperature dependence of the director’s tilt angle for (R) enantiomers. The inset enlarges the overlapping curves for better visibility only.

The temperature dependence of the tilt angle (Θ) for all (R) enantiomers is shown in Figure 8.

All enantiomers possess a very high tilt angle of the director in the smectic layers. The highest values of the optical tilt angle (Θ = 43.5° at low temperatures) were found for the monofluorinated enantiomer—**I.4.(HF) (R)**. The lowest Θ values (~40.0°) were found for the difluorinated enantiomer. With increasing temperature, the values of the tilt angle decrease; the highest tilt angle values and their temperature ranges are given in Table 4. Among the studied enantiomers, the unsubstituted enantiomer and the one substituted by a fluorine atom in the ortho position can be considered orthoconics [25,26].

### 3.5. Properties of Eutectic Mixtures

Two newly synthesized enantiomers and three previously synthesized (*R*) enantiomers [42,43] were used to formulate two antiferroelectric mixtures (denoted as W-446 and W-447); their compositions and phase transition temperatures from DSC measurements obtained from the heating cycle are given in Table 5. The composition of the eutectic mixture was calculated using the equations proposed by Le Chatelier, Schröder, and van Laar, according to Refs. [58,59,60,61].

Both designed mixtures possess the antiferroelectric phase in a wide temperature range. The mixture W-447 directly transitions from the anticlinic to the isotropic phase. Materials with the direct SmC_A_*−Iso phase transition usually generate high tilt values rapidly during phase formation; moreover, the layers do not change their spacing too much upon cooling. Hence, the shrinkage of the layer is minimal. Since no phase transitions occur during further cooling, the number of defects is limited, which is essential for applying these materials [60]. Despite the antiferroelectric phase, the mixture W-446 also possesses the ferroelectric phase in a medium temperature range. Both mixtures exhibit relatively low melting points. The clearing point is below 92 °C for the two-component mixture, W-447, and is slightly above 125 °C for the three-component one, W-446.

**Figure 9:**
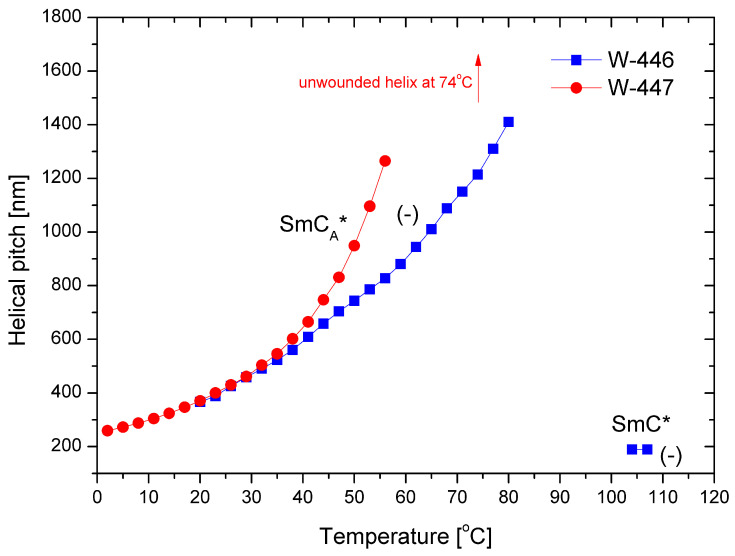
Temperature dependence of the helical pitch length for the mixtures W-446 and W-447; “-” indicates the left-handed helix.

The helical pitch versus temperature for both mixtures is presented in Figure 9. In the SmC_A_* phase, the helical pitch values for both mixtures are comparable at low temperatures. As the temperature increases, the pitch elongates, ultimately reaching complete unwinding at 74 °C for the mixture W-447. The pitch is notably short (i.e., below 200 nm) in the SmC* phase. Additionally, the helix is left-handed in both phases.

**Figure 10:**
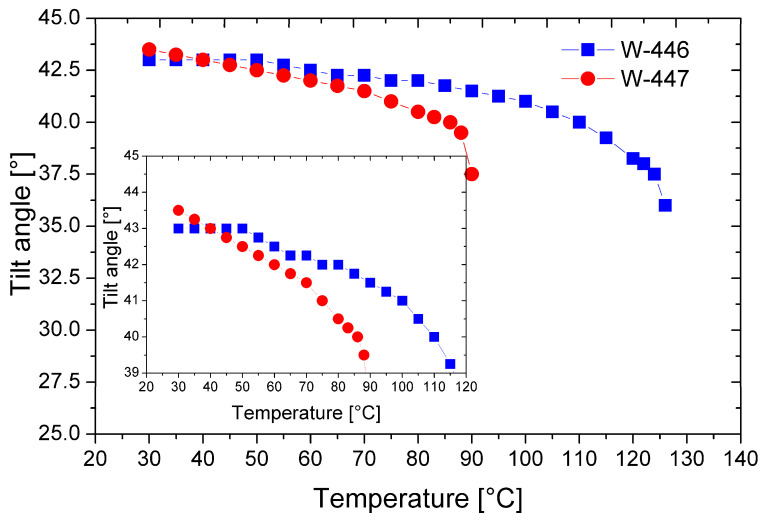
Temperature dependence of the tilt angle of the director for the mixtures W-446 and W-447. The inset enlarges the overlapping curves for better visibility only.

Figure 10 shows the temperature dependencies of the tilt angle of the director for the mixtures W-446 and W-447.

In both mixtures, a high tilt angle of the director is generated just below the phase transition from the isotropic to the smectic phase. As mentioned earlier, this is advantageous, as it minimizes the formation of chevron-type defects in surface-stabilized geometries. Notably, at temperatures below 50 °C, both mixtures exhibit a relatively high tilt angle of the director, exceeding 42 degrees. Furthermore, the mixture W-447 has a tilt angle above 43 degrees near room temperature, significantly enhancing the electro-optic effect’s contrast [26].

## 4. Conclusions

We have synthesized and characterized four liquid crystalline (*R*) enantiomers, each featuring a three-benzene-ring molecular core, a perfluoroalkoxyalkoxy terminal chain, complemented by a chiral center based on (*R*)-(−)-2-octanol. Their mesophase behavior, helical parameters, smectic layer thickness, the tilt angle of the director, and potential for utilization for the LC mixture formulation were systematically studied and evaluated. Two types of phase sequences are observed for the synthesized enantiomers. All enantiomers were found to be enantiotropic LCs and exhibit a broad temperature range of the antiferroelectric phase. The lowest melting points are observed for the monofluorinated esters. In the ferroelectric phase, the helix is left-handed, with almost temperature-independent and very short pitch values. In the antiferroelectric phase for the enantiomers **I.4.(FH) (*R*)** and **I.4.(HH) (*R*)**, the helix is left-handed, and the pitch increases with temperature. In the case of the enantiomers **I.4.(HF) (*R*)** and **I.4.(FF) (*R*)**, the values of helical pitch are above the measuring range of the used spectrophotometer. Most importantly, all the studied enantiomers exhibit the tilt angle of the director, exceeding 40.0° over a broad temperature range. In the case of the enantiomers **I.4.(HH) (*R*)** and **I.4.(HF) (*R*)**, the tilt angle values are sufficiently high to classify them as orthoconic AFLCs.

The antiferroelectric phase characterizes the eutectic mixtures W-446 and W-447, formulated using the designed enantiomers in a very broad temperature range, a long helical pitch, especially at higher temperatures, and they are near-orthoconic AFLCs. Furthermore, the favorable direct SmC_A_*−Iso phase transition was detected for one of the designed mixtures.

We can summarize that the synthesized (*R*) enantiomers and previously synthesized (*R*) enantiomers can be very useful for the formulation of LC eutectic mixtures (both binary and multicomponent) for further use in various devices (e.g., in photonics).

## Figures and Tables

**Table 1:** The acronyms and molecular ions for the synthesized enantiomers.

Acronym of the Enantiomer	Molecular Ion
**I.4.(HH) (*R*)**	701[M + H]^−^
**I.4.(HF) (*R*)**	718[M − H]^−^
**I.4.(FH) (*R*)**	719[M + H]^−^
**I.4.(FF) (*R*)**	735[M − H]^−^

**Table 2:** Relative peak areas of (*R*) enantiomers obtained by chiral HPLC and the corresponding enantiomeric excess values of the enantiomers.

Peak Area (%)	I.4.(HH) (*R*)	I.4.(HF) (*R*)	I.4.(FH) (*R*)	I.4.(FF) (*R*)
**(*S*)**	1.086	0.082	0.081	1.610
**(*R*)**	98.914	99.918	99.919	98.390
**% *ee***	97.83	99.84	99.84	96.78

**Table 3:** The phase sequences and phase transition temperatures (in the heating and cooling cycles) [°C] and phase transition enthalpies in brackets [J·g^−1^] for (*R*) enantiomers determined from DSC in the heating/cooling cycles with a rate of 5 K·min^−1^. The m.p is bolded, and the c.p. is italicized.

Acronym of the Enantiomer	Cr	T [ΔH]	SmC_A_*	T [ΔH]	SmC*	T [ΔH]	SmA*	T [ΔH]	Iso
**I.4.(HH) (*R*)** heating	•	75.4 [+20.9]	•	110.5 [+0.05]	•	*130.6* [+8.5]	–		•
**I.4.(HH) (*R*)** cooling	•	23.6 [−12.0]	•	104.1 [−0.06]	•	129.1 [−8.1]	–		•
**I.4.(HF) (*R*)** heating	•	48.7 [+28.8]	•	88.9 [+0.07]	•	*108.5* [+8.3]	–		•
**I.4.(HF) (*R*)** cooling	•	6.2 [−16.1]	•	83.5 [−0.07]	•	106.6 [−6.7]	–		•
**I.4.(FH) (*R*)** heating	•	68.4 [+34.6]	•	98.4 [+0.07]	•	115.5 [+0.8]	•	*116.5* [+7.5]	•
**I.4.(FH) (*R*)** cooling	•	40.6 [−25.9]	•	92.5 [−0.07]	•	114.2 [−0.7]	•	114.8 [−7.7]	•
**I.4.(FF) (*R*)** heating	•	76.5 [+35.7]	•	98.3 [+0.07]	•	*115.8* [+7.2]	–		•
**I.4.(FF) (*R*)** cooling	•	56.0 [−33.4]	•	93.9 [−0.06]	•	114.3 [−6.4]	–		•

**Table 4:** The highest tilt angle for (*R*) enantiomers.

Acronym of the Enantiomer	Θ [°]	T [°C]
**I.4.(HH) (*R*)**	~43.0	30–75
**I.4.(HF) (*R*)**	~44.0	25–35
**I.4.(FH) (*R*)**	~41.0	45–75
**I.4.(FF) (*R*)**	~40.0	75–90

**Table 5:** The composition and phase transition temperatures [°C] determined on heating for the formulated LC mixtures.

Mixture/Phase Transition Temperatures	Acronym of the Enantiomer	Weight Ratio [%]
**W-446**/Cr 44.1 SmC_A_* 105.4 SmC* 126.0 Iso	**I.4.(HH) (*R*)**	26.34
	**I.2.(HH) (*R*)**	35.58
	**I.6.(HH) (*R*)**	38.08
**W-447**/Cr 27.3 Cr′ 32.9 SmC_A_* 91.8 Iso	**I.4.(HF) (*R*)**	35.59
	**I.3.(HF) (*R*)**	64.41

## Data Availability

The original contributions presented in the study are included in the article and Supplementary Materials, further inquiries can be directed to the corresponding author.

## Supplementary Materials

The following supporting information can be downloaded at: https://www.mdpi.com/article/10.3390/ma17204967/s1, Figure S1: The mass spectrum and purity curve of the enantiomer denoted as **I.4.(HH) (*R*)**; Figure S2: The mass spectrum and purity curve of the enantiomer denoted as **I.4.(HF) (*R*)**; Figure S3: The mass spectrum and purity curve of the enantiomer denoted as **I.4.(FH) (*R*)**; Figure S4: The mass spectrum and purity curve of the enantiomer denoted as **I.4.(FF) (*R*)**.
