# Supplementary material for: Synthesis and Properties of Highly Tilted Antiferroelectric Liquid Crystalline (R) Enantiomers
Source: Materials (Basel). 2024 Oct 11;17(20):4967. doi: 10.3390/ma17204967 (PMC11509236; doi:10.3390/ma17204967)
Supplement: Supplementary file 1 [file materials-17-04967-s001.zip › materials-3225550-Supplementary Materials.pdf]

Supplementary materials for the article

Synthesis and Properties of Highly Tilted Antiferroelectric Liquid Crystalline (*R*) Enantiomers

Magdalena Urbańska [1]*, Monika, Zając [1], Michał Czerwiński [1], Przemysław Morawiak [2], Alexej Bubnov [3], Aleksandra Deptuch [4]

[1] *Institute of Chemistry, Military University of Technology, ul. Sylwestra Kaliskiego 2, 00-908 Warsaw, Poland; monika.zajac@wat.edu.pl (M.Z.); michal.czerwinski@wat.edu.pl (M.C.)*

[2] *Institute of Applied Physics, Military University of Technology, ul. Sylwestra Kaliskiego 2, 00-908 Warsaw, Poland; przemyslaw.morawiak@wat.edu.pl*

[3] *Institute of Physics of the Czech Academy of Sciences, Na Slovance 2, 18200 Prague, Czech Republic; bubnov@fzu.cz*

[4] *Institute of Nuclear Physics Polish Academy of Sciences, Radzikowskiego 152, 31-342 Krakow, Poland; aleksandra.deptuch@ifj.edu.pl*

* *Corresponding author: Magdalena Urbańska, e-mail: magdalena.urbanska@wat.edu.pl*

PREPARATIVE RECIPE FOR THE SYNTHESIS OF (*R*)-4′-(1-METHYLHEPTYLOXY-CARBONYL)BIPHENYL-4-YL 4-[4-(2,2,3,3,4,4,4-HEPTAFLUOROBUTOXY)BUT-1-OXY]BENZOATE - I.4.(HH) (*R*)

To a suspension of 2.94 g (7.5 mmol) 4-[4-(2,2,3,3,4,4,4-heptafluorobutoxy)but-1-oxy]benzoic acid in dry toluene (~70 mL) was added 0.75 mL (8.25 mmol) oxalyl chloride and one drop of N,N-dimethylformamide, after which a Vigorous reaction occurred. After the gas evolution had ceased, the mixture was heated at 30°C under stirring for 4 hours. The clear solution was heated to reflux, and the excess oxalyl chloride was distilled with toluene (~15 mL) using a Vigreux column. Then 2.44 g (7.5 mmol) (*R*)-4-hydroxy-4′-(1-methylheptylcarbonyl)biphenyl and 1.2 mL (15 mmol) of pyridine were added to the cold solution. The mixture was stirred at 60°C for 16 hours, then cooled to room temperature and poured into the solution prepared from concentrated hydrochloric acid (~1 mL) and water (~100 mL). The layers were separated, and the organic layer was washed twice with water and passed through activated carbon. Then, it was dried over anhydrous magnesium sulfate, and the solvent was evaporated to dryness. The final product was dissolved in methylene chloride, purified by column chromatography on silica gel, and crystallized from ethanol in anhydrous form. The remaining enantiomers: (*R*)-4′-(1-methylheptyloxycarbonyl)biphenyl-4-yl 4-[4-(2,2,3,3,4,4,4-heptafluorobutoxy)-but-1-oxy]-2-fluorobenzoate - **I.4.(HF) (*R*)**, (*R*)-4′-(1-methylheptyloxycarbonyl)biphenyl-4-yl 4-[4-(2,2,3,3,4,4,4-heptafluorobutoxy)but-1-oxy]-3-fluorobenzoate - **I.4.(FH) (*R*)** and (*R*)-4′-(1-methyl-heptyloxycarbonyl)biphenyl-4-yl 4-[4-(2,2,3,3,4,4,4-heptafluorobutoxy)but-1-oxy]-2,3-difluorobenzoate - **I.4.(FF) (*R*)** were synthesized in the same manner.

MASS SPECTRA AND PURITY CURVES OF (*R*) ENANTIOMERS

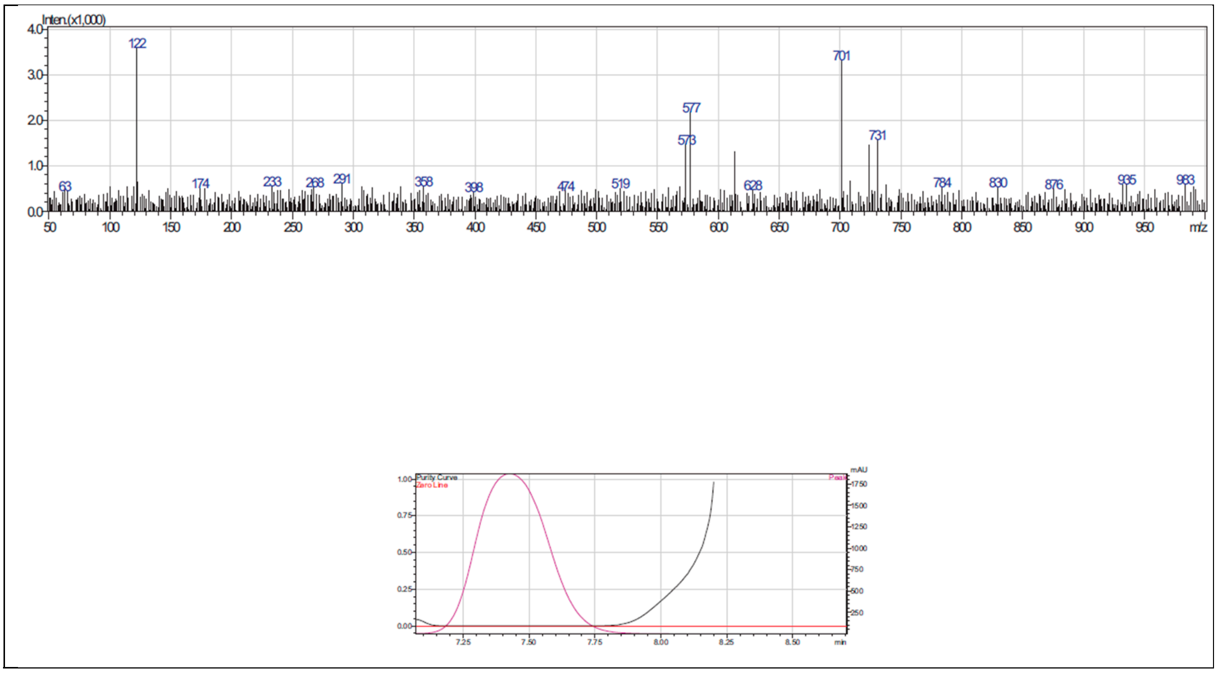

Figure S1. The mass spectrum and purity curve of the enantiomer denoted as **I.4.(HH) (*R*)**.

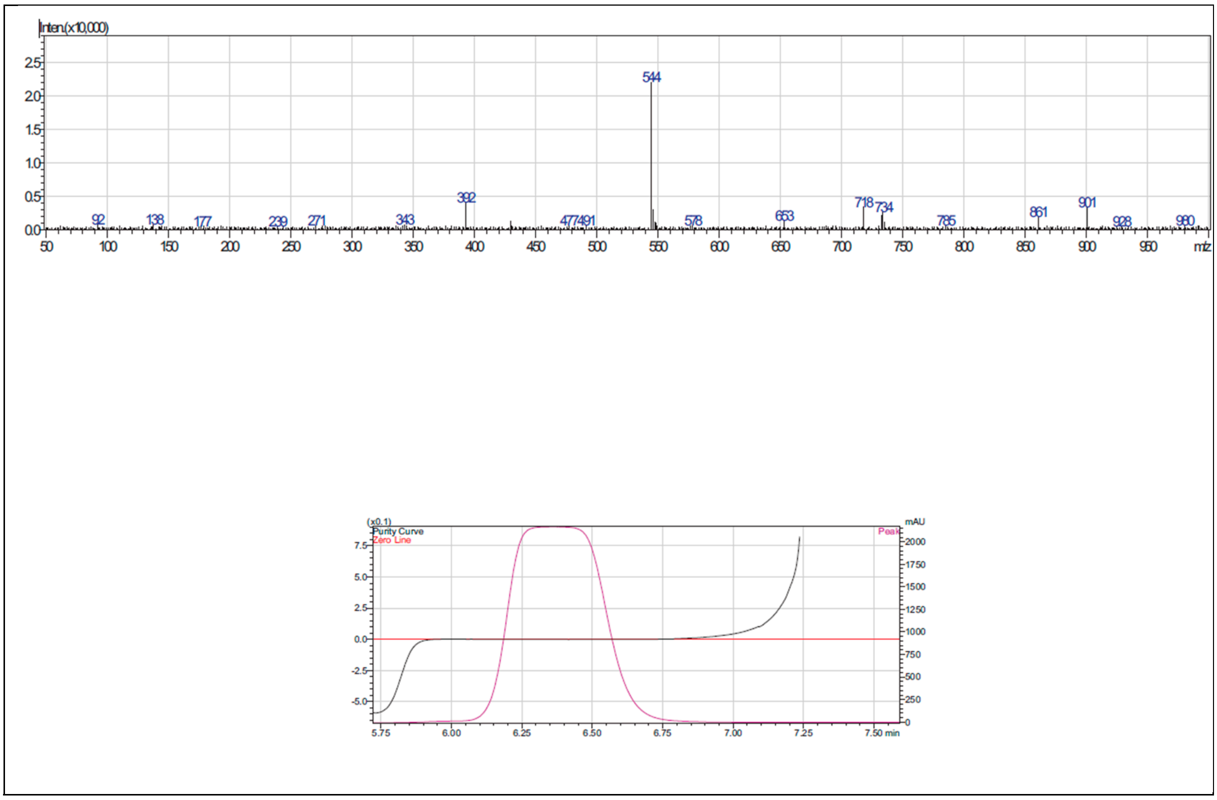

Figure S2. The mass spectrum and purity curve of the enantiomer denoted as **I.4.(HF) (*R*)**.

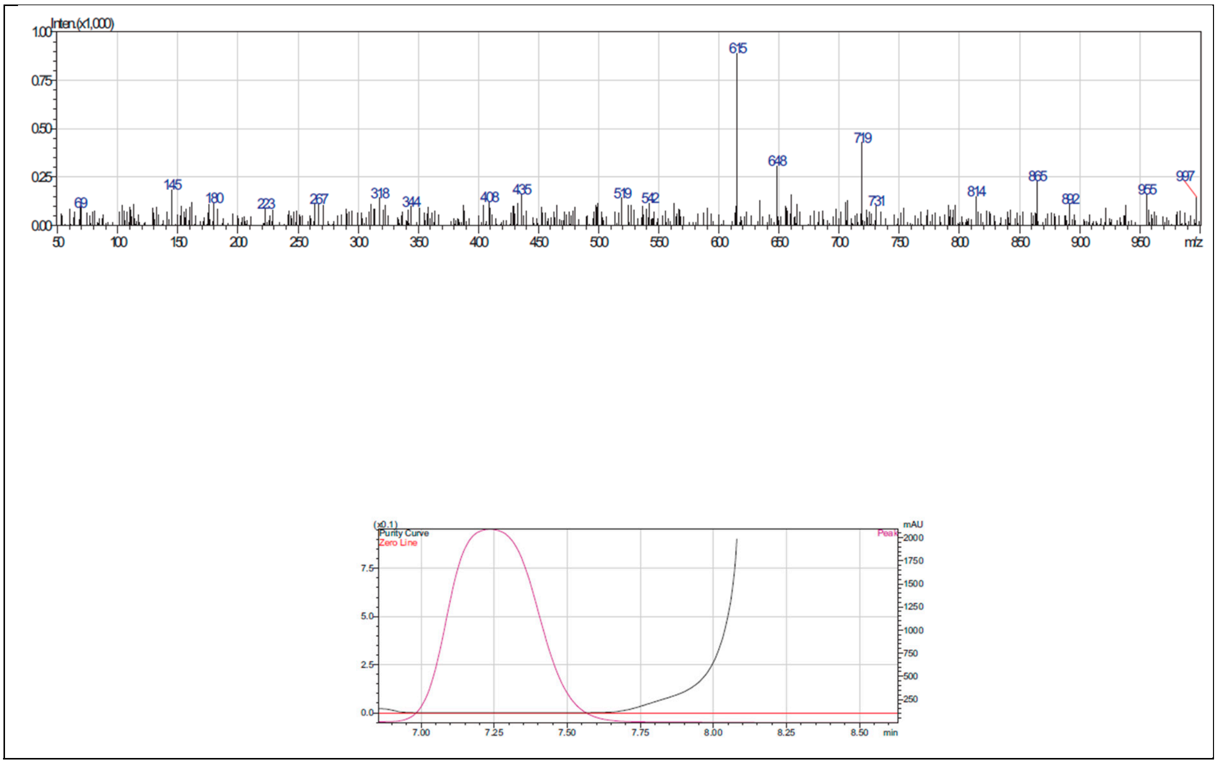

Figure S3. The mass spectrum and purity curve of the enantiomer denoted as **I.4.(FH)** **(*R*)**.

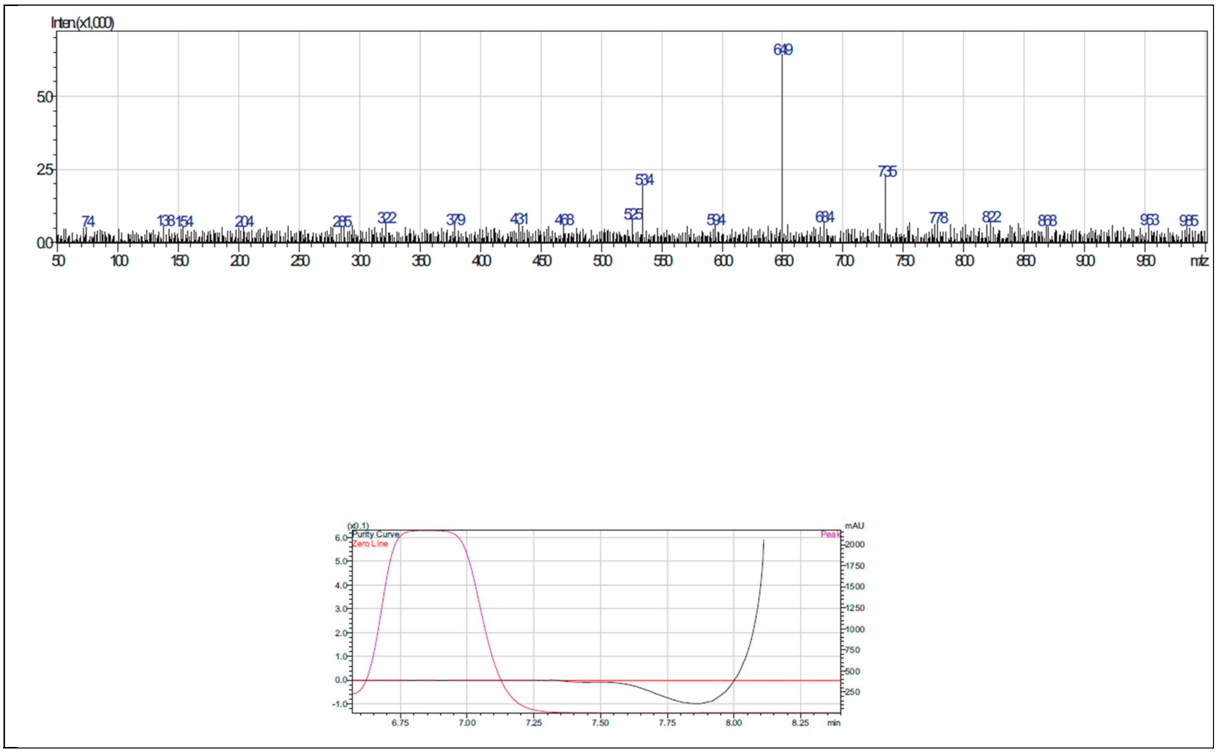

Figure S4. The mass spectrum and purity curve of the enantiomer denoted as **I.4.(FF)** **(*R*)**.
